# Decomposing socio-economic inequalities in leisure-time physical inactivity: the case of Spanish children

**DOI:** 10.1186/s12939-016-0394-9

**Published:** 2016-07-12

**Authors:** Eduardo Gonzalo-Almorox, Rosa M. Urbanos-Garrido

**Affiliations:** Institute of Health and Society, Newcastle University, ᅟNewcastle, ᅟUnited Kingdom; Department of Public Finance, School of Economics, Complutense University of Madrid, Campus de Somosaguas s/n, 28223 Pozuelo de Alarcón, Spain

**Keywords:** Health inequalities, Physical inactivity, Children, Spain, H51, I14, I18

## Abstract

**Background:**

Physical inactivity is associated with an increased risk of all-cause mortality and entails a substantial economic burden for health systems. Also, the analysis of inequality in lifestyles for young populations may contribute to reduce health inequalities during adulthood. This paper examines the income-related inequality regarding leisure-time physical inactivity in Spanish children.

**Methods:**

In this cross-sectional study based on the Spanish National Health Survey for 2011-12, concentration indices are estimated to measure socioeconomic inequalities in leisure-time physical inactivity. A decomposition analysis is performed to determine the factors that explain income-related inequalities.

**Results:**

There is a significant socioeconomic gradient favouring the better-off associated with leisure-time physical inactivity amongst Spanish children, which is more pronounced in the case of girls. Income shows the highest contribution to total inequality, followed by education of the head of the household. The contribution of several factors (education, place of residence, age) significantly differs by gender.

**Conclusions:**

There is an important inequity in the distribution of leisure-time physical inactivity. Public policies aimed at promoting physical activity for children should prioritize the action into the most disadvantaged subgroups of the population. As the influence of determinants of health styles significantly differ by gender, this study points out the need of addressing the research on income-related inequalities in health habits from a gender perspective.

## Background

Physical inactivity is a public health concern worldwide. The WHO regards physical inactivity as the fourth leading risk factor for mortality, causing about 6 % of deaths globally [1]. It is also closely associated to a more frequent use of healthcare resources [2] and to several chronic diseases that increase health care expenditure [3], including coronary heart disease, colon cancer, hypertension, stroke, breast cancer, type 2 diabetes and osteoporosis [4]. In young people, appropriate levels of physical activity contribute to the development of healthy musculoskeletal tissues, a healthy cardiovascular system and neuromuscular awareness; it also facilitates the maintenance of a healthy body weight and has been associated with psychological benefits [5].

The relationship between the socioeconomic background of individuals and their recreational physical activity has been addressed in the literature [6–10]. Empirical evidence indicates that propensity to engage with physical activity depends positively on income and education [6–9], even when controlling for local area deprivation, the availability of physical recreation and sporting facilities and the local weather [9]. While income helps to overcome the financial barriers for accessing gyms or sport clubs [6], education seems to be related to a better understanding of the health benefits of physical activity [6, 7]. Furthermore, the educational gradient appears more intense in women [10]. The socioeconomic gradient in physical activity in younger populations has also been documented. Income, socioeconomic status and parental education appear to be positively correlated to high physical levels for children and adolescents [11–16]. In Spain, Rey-López et al. [16] have shown socioeconomic factors such as parental education prove to be inversely associated with sedentary behaviours in adolescents that include TV viewing or computer and videogames use during weekdays, which may be considered as substitute activities for physical exercise [16].

This paper represents a step forward in this literature, as it explicitly estimates the degree of income-related inequality in leisure-time physical inactivity for Spanish children by employing the approach proposed by Wagstaff et al. [17], and decomposes inequality in order to calculate the relative contribution of each explanatory factor. The adoption of this approach has important policy implications since it contributes to improve the design of actions to engage children with physical activity and to cope with inequalities. The analysis of inequality in lifestyles and its determinants for young populations can be useful to guide public interventions in order to reduce, not only current, but also future health inequalities, since habits during childhood shape future habits [18, 19], and hence social inequalities in childhood tend to be reproduced in the adult population. An additional contribution of this paper resides in the use of the Spanish National Health Survey, a more comprehensive dataset than those used in prior studies, therefore producing more robust estimates of inequality.

The objective of this research is then to quantify income-related inequality regarding leisure-time physical inactivity in Spanish children, and to identify the determinants of inequality. Previous literature has shown how physical activity habits, as well as perceptions about physical exercise, differ by gender [20, 21]. Therefore, we analyse boys and girls separately and examine whether the income-related inequality has also a gender component.

## Methods

### Data

The data used for this research are cross-sectional and come from the Spanish National Health Survey (SNHS). This is a representative survey of the Spanish population, carried out by the Spanish Institute of National Statistics (INE) and coordinated by the Ministry of Public Health, Social Services and Equality. The sample is stratified according to a three stages design. Primary strata are the regions and subsequent stages comprise census sections and main family dwellings. The final sample size of the SNHS is composed by about 24,000 homes distributed among 2000 census sections. Data were collected throughout interviews conducted from July 2011 to June 2012 at a national level. The main objective of the survey is to obtain data about population health and health care use, and also about its determining factors. The SNHS is structured in three different types of questionnaire: household, children (0–14 years old) and adults questionnaire (15 or over). Data on children are collected via interviews with one of the adults in the home (usually one of the parents −94 %-, although the questionnaire may be answered by other adult –e.g. grandparent or older sibling-). Considering the purposes of the research, we chose the children sample of SNHS, containing information about boys and girls from 0 to 14 years old. The complete dataset used for this analysis comprised socioeconomic data extracted from the household survey and data about physical activity and other health characteristics extracted from the child-related dataset.

### Definition of variables

Our variable of interest is based on the information provided by the SNHS about the frequency of leisure time physical activity. The survey categorizes the responses into four groups: ‘never’, ‘occasionally’, ‘several times monthly’ or ‘several times weekly’. We created our dependent variable by dichotomizing these data into two categories: *no activity* (‘never’) vs. *some activity* (occasionally and several times monthly/weekly).

Measurement of inequality through a concentration index and decomposition analysis requires a continuous variable that enables to rank individuals according to their socioeconomic status. However, the SNHS presents the information about the net monthly income corresponding to each household as a categorical variable with ten response intervals. Following Costa-i-Font & Gil [22], we estimated a continuous variable of the income by employing the predictions of an interval regression model based on the information of the head of the household, where the covariates include gender, age, education, labour status, social class and region of residence. Results from the estimation are shown in the Appendix. The modified OECD equivalence scale was used to calculate equivalent income.

The explanatory variables are divided into five groups. According to Roberts et al. [23], besides individual factors, behaviour may be influenced by social and cultural environment, economic environment, education and physical environment. As individual factors we include age, which is divided into three groups (under 5, 5–9 and 10–14 years old), as well as the presence of any physical limitation for developing normal activity during the last six months. We also include some variables provided by the SNHS that represent children’s mental health in five different dimensions: emotional symptoms, behaviour problems, hyperactivity, problems with their peers and pro-social behaviour. These five indicators range from 0 to 10, where 0 represents best mental health and 10 is the worst mental health possible except for the last dimension (pro-social behaviour), where 10 indicates the best mental health status. As the battery of questions used to construct these indicators only applies to children aged 4 or over, our sample is consequently restricted to children from 4 to 14 years old. A dummy variable that represents whether the child is Spanish or foreigner is used to capture the cultural environment. The economic environment is proxied by the logarithm of total monthly net equivalent income of the household, and education is represented by a dummy which reflects whether the head of the household has university education or equivalent studies. We have reduced the number of educational variables, since this information is already included in the estimation of the household income. Physical environment is firstly proxied by the population size of the area where children live (under 10,000 people vs. rest of areas), as bigger settlements could discourage transport-related physical activity (walking and cycling), an also may be associated to disincentives for children’s physical activity derived from fear of traffic or from a less children-friendly environment [23]. Secondly, we included the region of residence. Spanish regions have political competences in different public policies such as health, education, culture or recreational activities. Different regions may therefore have different programs of health promotion among children, and also different density of sports and recreational facilities, so it is important to capture these potential disparities.

### Methods for measuring and decomposing inequality

We used conventional methods for measuring and decomposing income-related inequality associated with physical inactivity. In particular, in order to estimate inequality we employ the concentration index as proposed by Kakwani [24] and Wagstaff et al. [25]. The concentration index (*CI*) can be calculated as follows:

1 $$ CI=\frac{2}{\overline{y}}\operatorname{cov}\left({y}_i,{r}_i\right) $$

where $\overline{y}$ is the mean of the ill-health proxy (in our case, the average rate of leisure-time physical inactivity), and r_i_ is the cumulative percentage that each individual represents over the total population once the latter has been ranked by income. The values of this index range from −1 to 1, or from $\overline{y}-1$ to $1-\overline{y}$ when the variable indicating ill-health is dichotomous [26]. A negative (positive) index shows that ill-health is concentrated in individuals with relatively low (high) income. If *CI* = 0, then there is no income-related inequality in the distribution of ill-health.

When the variable *y* is dichotomous, Erreygers [27] suggests the following correction in the concentration index, in order to allow comparisons between groups of individuals that may present different levels of average ill-health:

2 $$ E(y)=\frac{4\overline{y}}{y^{\max }-{y}^{\min }}CI $$

where *y*^max^ and *y*^min^ are the extremes of the health variable.

Following Wagstaff et al. [17], when there is a linear relationship between *y* and a set of k explanatory variables *x*, CI may be expressed as a weighted sum of the partial concentration indices for the explanatory factors of inequality, being the weight the ill-health elasticity with respect to each of the *x*_k_ variables [17]:

3 $$ CI={\displaystyle \sum_k\left(\frac{\beta_k{\overline{x}}_k}{\overline{y}}\right)}\kern0.5em C{I}_k+\frac{GC{I}_e}{\overline{y}} $$

where ${\overline{x}}_k$ is the mean of the variable representing ill-health, *CI*_*k*_ are the partial concentration indices for the k explanatory factors considered and *GCI*_*e*_ is the generalized concentration index for the error term. When the dependent variable is a dummy, the estimation may be carried out by maximum likelihood, and decomposition analysis will be possible if some linear approximation to the non-linear model is made [28]. In our case, the decomposition of inequality will be conducted using a probit model. The *CI* can then be rewritten as:

4 $$ CI={\displaystyle \sum_k\left(\frac{\beta_k^m{\overline{x}}_k}{\overline{y}}\right)}\kern0.5em C{I}_k+\frac{GC{I}_{\varepsilon }}{\overline{y}} $$

where *β*_*k*_^*m*^ are the partial effects (*dy*/*dx*_*k*_) evaluated at sample means, and *GCIe* is the error term.

Therefore, this method aims at disentangling the different sources of income related-inequality and allows to remove the effect of those variables which are considered irrelevant for policy [29].]. If the Erreygers’ corrected index is used, the decomposition of inequality may be expressed by (5) [30], although it provides the same decomposition results as (4).

5 $$ E=4\cdot {\displaystyle \sum_k\left({\beta}_k^m{\overline{x}}_k\right)}\kern0.5em C{I}_k+GC{I}_{\varepsilon } $$

The statistical analysis was performed using StataSE 13 ©. The concentration indices (CI and E) were calculated by using the conindex command [31].

## Results

Table 1 shows the definition of variables and the descriptive statistics for the sample of girls and boys. We use 4662 of the total 5495 observations that compose the sample of children (2460 for boys and 2219 for girls), after removing those missing observations of our dependent variable and the unknown response. All the calculations have been made by using the sampling weights.

**Table 1:** Definition of variables and descriptive statistics of the sample

		Boys (*n* = 2460)				Girls (*n* = 2219)			
Variable	Definition	Mean^a^	Std. Dev.	Min	Max	Mean^a^	Std. Dev.	Min	Max
Physical_inactivity	1 if the child does not make physical exercise in her/his leisure time; 0 otherwise	16.8 %	0.374	0	1	24.2 %	0.428	0	1
Ageunder5	1 if aged under 5; 0 otherwise (omitted category)	29.2 %	0.457	0	1	28.5 %	0.454	0	1
Age5to9	1 if aged 5–9; 0 otherwise	36.4 %	0.481	0	1	35.6 %	0.479	0	1
Age10to14	1 if aged 10–14; 0 otherwise	34.4 %	0.475	0	1	35.9 %	0.480	0	1
Limit_phys	1 if physical problems limiting normal activity; 0 otherwise	6.4 %	0.244	0	1	6.2 %	0.242	0	1
Emotional_symtoms	Score for emotional symptoms (from 0 –best- to 10 –worst)	1.318	1.733	0	10	1.430	1.844	0	10
Behaviour_problems	Score for behaviour problems (from 0 –best- to 10 –worst-)	1.320	1.617	0	10	1.288	1.622	0	9
Hyperactivity	Score for hyperactivity (from 0 –best- to 10 –worst-)	3.392	2.924	0	10	2.890	2.696	0	10
Problems with peers	Score for problems with peers (from 0 –best- to 10 –worst-)	0.902	1.410	0	10	0.800	1.299	0	9
Pro-social behaviour	Score for pro-social behavior (from 0 –worst- to 10 –best-)	6.975	3.856	0	10	7.118	3.887	0	10
Foreigner	1 if foreigner; 0 if Spanish	8.4 %	0.278	0	1	8.9 %	0.285	0	1
Log_income	Logarithm of total monthly net equivalent income	6.573	0.527	1.775	7.684	6.572	0.545	2.993	7.771
Univ_educ	1 if the head of the household has university education; 0 otherwise	21.1 %	0.408	0	1	24.0 %	0.427	0	1
Rural	1 if place of residence < 10.000 inhabitants; 0 otherwise	20.6 %	0.405	0	1	18.4 %	0.387	0	1
Region_1	1 if resident in Andalusia; 0 otherwise	19.9 %	0.340	0	1	19.8 %	0.397	0	1
Region_2	1 if resident in Aragon; 0 otherwise	2.4 %	0.152	0	1	2.8 %	0.164	0	1
Region_3	1 if resident in Asturias; 0 otherwise	1.8 %	0.132	0	1	1.7 %	0.129	0	1
Region_4	1 if resident in Balearic Is.; 0 otherwise	2.5 %	0.156	0	1	2.4 %	0.154	0	1
Region_5	1 if resident in Canary Is.; 0 otherwise	4.0 %	0.196	0	1	3.9 %	0.195	0	1
Region_6	1 if resident in Cantabria; 0 otherwise	1.0 %	0.102	0	1	1.1 %	0.104	0	1
Region_7	1 if resident in Castilla-Leon; 0 otherwise	4.6 %	0.209	0	1	4.5 %	0.207	0	1
Region_8	1 if resident in Castilla-La Mancha; 0 otherwise	4.3 %	0.203	0	1	4.5 %	0.208	0	1
Region_9	1 if resident in Catalonia; 0 otherwise	15.5 %	0.362	0	1	16.0 %	0.366	0	1
Region_10	1 if resident in Valencia; 0 otherwise	11.6 %	0.320	0	1	11.4 %	0.318	0	1
Region_11	1 if resident in Extremadura; 0 otherwise	2.1 %	0.143	0	1	2.3 %	0.149	0	1
Region_12	1 if resident in Galicia; 0 otherwise	4.5 %	0.207	0	1	4.8 %	0.214	0	1
Region_13	1 if resident in Madrid; 0 otherwise	15.3 %	0.360	0	1	14.2 %	0.349	0	1
Region_14	1 if resident in Murcia; 0 otherwise	3.8 %	0.191	0	1	3.7 %	0.190	0	1
Region_15	1 if resident in Navarre; 0 otherwise	1.5 %	0.121	0	1	1.4 %	0.117	0	1
Region_16	1 if resident in Basque C.; 0 otherwise	4.1 %	0.198	0	1	4.4 %	0.205	0	1
Region_17	1 if resident in La Rioja; 0 otherwise	0.7 %	0.083	0	1	0.7 %	0.081	0	1
Region_18	1 if resident in Ceuta and Melilla; 0 otherwise	0.4 %	0.064	0	1	0.4 %	0.067	0	1

^a^Mean can be expressed in percentage only for dummies, to reflect the proportion of individuals with value 1

As it is shown in Table 1, leisure-time physical inactivity is more prevalent among girls (24.2 %) than among boys (16.8 %). Although there are no gender differences in the presence of physical limitations, girls mostly show better mental health than boys, particularly in those dimensions linked to hyperactivity and problems with peers.

Table 2 shows the concentration indices for leisure-time physical inactivity, both standard CI and the correction proposed by Erreygers. Our results suggest that income-related inequality is statistically significant and favours the better-off. Also, the concentration indices point out that inequality is higher for girls than for boys.

**Table 2:** Concentration indices for leisure-time physical inactivity

	Boys	Girls
Standard CI	−0.078 [0.000]	−0.090 [0.000]
Erreygers CI	−0.052 [0.007]	−0.087 [0.003]

*p*-value in brackets

Table 3 reports the Erreygers CI decomposition by gender. It firstly shows the partial effects obtained by the probit estimation, evaluated at sample means. The second column indicates the elasticity of physical inactivity for each explanatory variable. The third column displays the partial concentration index (also corrected by the Erreygers normalization) for each of these determinants. A negative (positive) sign indicates that the variable has a pro-poor (pro-rich) distribution. Finally, the last two columns of Table 3 reports, respectively, the absolute and percentage contributions to overall income-related inequality. The absolute contribution is the product of the elasticity and the partial concentration index for each factor, so it will depend both on the impact of each variable on leisure-time physical inactivity and on its unequal distribution by income. A negative (positive) absolute contribution implies that, if inequality in leisure-time physical inactivity was determined by that variable alone, then it would favour the better-off (worse-off). The percentage contribution is obtained by dividing the absolute contribution by the overall income-related inequality, as measured by Erreygers CI.

**Table 3:** Contributions to inequality in physical inactivity

	Boys					Girls				
Variable	Partial Effect	Elasticity	Conc.Index	Contribution	% Contribution	Partial Effect	Elasticity	Conc.Index	Contribution	% Contribution
Age5to9	−0.100***	−0.2151	0.0140	−0.0030	5.77 %	−0.141***	−0.2068	0.0229	−0.0047	5.42 %
Age10to14	−0.121***	−0.2461	−0.0302	0.0074	−14.23 %	−0.063	−0.0932	−0.0217	0.0020	−2.32 %
Limit_phys	0.090**	0.0348	0.0098	0.0003	−0.65 %	0.144***	0.0370	0.0291	0.0011	−1.23 %
Emotional_symptoms	0.007	0.0542	−0.0227	−0.0012	2.36 %	0.020***	0.1154	−0.0549	−0.0063	7.26 %
Behaviour_problems	−0.009	−0.0727	−0.0330	0.0024	−4.60 %	−0.011	−0.0572	−0.0975	0.0056	−6.38 %
Hyperactivity	−0.004	−0.0792	−0.0280	0.0022	−4.25 %	0.003	0.0299	−0.0456	−0.0014	1.56 %
Problems_with_peers	0.021***	0.1126	−0.0542	−0.0061	11.68 %	0.020**	0.0652	−0.0747	−0.0049	5.58 %
Pro-social_behaviour	−0.018***	−0.7571	−0.0033	0.0025	−4.77 %	−0.028***	−0.8142	−0.0068	0.0055	−6.33 %
Foreigner	0.035	0.0179	−0.1600	−0.0029	5.48 %	0.109**	0.0420	−0.2126	−0.0089	10.23 %
Log_income	−0.020	−0.7727	0.0280	−0.0216	41.42 %	−0.051**	−1.3836	0.0423	−0.0585	66.98 %
Univ_educ	−0.039*	−0.0499	0.4057	−0.0202	38.74 %	−0.030	−0.0300	0.5937	−0.0178	20.42 %
Rural	−0.010	−0.0125	−0.0331	0.0004	−0.79 %	−0.035	−0.0268	−0.0515	0.0014	−1.58 %
Region_2	0.069	0.0096	0.2572	0.0025	−4.74 %	−0.050	−0.0057	0.2614	−0.0015	1.70 %
Region_3	0.187***	0.0196	0.0491	0.0010	−1.84 %	0.006	0.0004	0.1809	0.0001	−0.08 %
Region_4	0.159	0.0239	−0.0623	−0.0015	2.86 %	0.040	0.0040	0.0976	0.0004	−0.45 %
Region_5	0.039	0.0095	−0.2658	−0.0025	4.85 %	−0.160***	−0.0269	−0.2869	0.0077	−8.86 %
Region_6	0.397	0.0255	0.0165	0.0004	−0.80 %	0.285***	0.0128	−0.0415	−0.0005	0.61 %
Region_7	−0.016	−0.0044	−0.1514	0.0007	−1.29 %	−0.117***	−0.0215	−0.2152	0.0046	−5.30 %
Region_8	−0.003	−0.0008	0.1042	−0.0001	0.15 %	−0.050	−0.0093	0.1065	−0.0010	1.14 %
Region_9	0.080**	0.0737	0.1126	0.0083	−15.88 %	−0.081**	−0.0531	0.1528	−0.0081	9.30 %
Region_10	0.007	0.0045	−0.1042	−0.0005	0.90 %	−0.047	−0.0220	−0.1229	0.0027	−3.10 %
Region_11	−0.003	−0.0004	−0.0847	0.0000	−0.06 %	−0.098**	−0.0091	−0.1829	0.0017	−1.92 %
Region_12	0.002	0.0006	−0.0560	0.0000	0.06 %	−0.037	−0.0074	−0.0817	0.0006	−0.69 %
Region_13	−0.016	−0.0143	0.1578	−0.0023	4.33 %	−0.064*	−0.0388	0.2148	−0.0083	9.54 %
Region_14	0.081	0.0182	−0.0538	−0.0010	1.88 %	0.094*	0.0145	−0.1585	−0.0023	2.63 %
Region_15	0.103**	0.0091	0.1865	0.0017	−3.25 %	0.085	0.0048	0.4073	0.0020	−2.24 %
Region_16	−0.093***	−0.0229	0.3038	−0.0070	13.31 %	−0.170***	−0.0312	0.3602	−0.0112	12.87 %
Region_17	0.002	0.0001	0.0819	0.0000	−0.01 %	−0.119**	−0.0032	0.0923	−0.0003	0.34 %
Region_18	0.132**	0.0032	−0.3516	−0.0011	2.16 %	−0.001	0.0000	−0.3763	0.0000	0.00 %
Residual				−0.0111	21.25 %				0.0132	−15.08 %

^*^ *p* < 0.10. ^**^ *p* < 0.05. ^***^ *p* < 0.01

Our results suggest that physical inactivity in leisure time is significantly associated with age, particularly for boys. Hence, children under 5 are most likely to be inactive. As expected, inactivity tends to increase when physical limitations are present, and also when children show mental health problems. However, the results show some differences according to sex: for boys, the effect is only significant for mental health problems related to the presence of difficulties in the relationship with peers and to the presence of shortcomings in pro-social behaviour (such as sharing or bringing support). Since low values for the pro-social behaviour dummy represent problems in this dimension of mental health, the negative sign of the partial effect indicates that a higher score on pro-social behaviour diminishes the probability of being physical inactive in leisure-time. In contrast, leisure-time physical inactivity in girls seems to be also influenced by the presence of emotional symptoms (being worried, unhappy, no self-confident, scary; or nervous before new situations). Those children who were born out of Spain are also more prone to be physically inactive than Spanish children, although the effect of nationality is only significant for girls.

Moreover, income and education have the expected signs, as they show a negative influence on the likelihood of experiencing physical inactivity in leisure time. Having a parent with university studies decreases the probability of being physical inactive by 0.039 and 0.03 for boys and girls, respectively. Also, a one per cent increase in the household income decreases the probability of showing physical inactivity by 0.02 for boys and 0.051 for girls. Nevertheless, the household income appears to be statistically significant only for girls, and education only for boys. Finally, with respect to the physical environment proxies, being resident in a small place does not seem to be associated to leisure-time physical inactivity. Meanwhile, regional dummies show quite different effects among boys and girls.

According to our results, inequality in physical activity is mainly explained by the direct effect of income, which accounts for 66.98 % of the girls’ concentration index, and 41.42 % in the case of boys. This fact is due to the larger elasticity of physical activity with respect to income in the girls’ sample, as it can be seen at Table 3. The second determinant of inequality appears to be education of the head of the household. In this case, the contribution to the leisure-time physical inactivity concentration index is higher for boys (38.74 %) than for girls (20.42 %). Again, this is caused by the different elasticity values for both samples. As it is shown at Table 3, there is little sensitivity of girls’ physical inactivity to educational level of the head of the household. Both income and education exhibit a negative absolute contribution to the leisure-time physical inactivity concentration index, what implies that they tend to favour the better-off.

Interestingly, when the effects of the regional variables are added up, the whole contribution of the place of residence to inequality is unimportant for boys (1.82 %), but significant for girls (13.91 %). The largest effect corresponds to the Basque country and it is quite similar for both sexes. This is due to the combination of two factors: the high concentration index for this regional dummy (indicating that the Basque country is one of the richest regions in Spain), and the high elasticity of physical inactivity to this variable. Children living in the Basque country show the lowest probability of being physically inactive, compared to those living in Andalusia. Similar results are obtained for Madrid. In general, physical inactivity seems to be less related to the place of residence for boys than for girls. However, regional results do not seem to be systematically related to regional income, geographic location or political sign. Therefore, disentangling regional effects would deserve further research.

Moreover, the contribution of nationality is higher for girls than for boys (10.23 % vs. 5.48 %, respectively), and in both cases tend to favour the better-off. This results from the combination of positive elasticities and negative concentration indices, thus indicating that migration status is relatively concentrated on poor people. Differences in the contribution of age by gender have to do with the dummy *Age10to14*, which is significant only in the boys’ model and indicates that oldest boys are less prone to be physically inactive than the youngest. Furthermore, the mental health variables, particularly when considered as an aggregate, have minor relevance in the explanation of inequality for both boys and girls. It has to be taken into account, however, that among the mental health dimensions problems with peers are the largest contributor to inequality in boys, while in girls it is to have emotional symptoms. Both factors tend to favour the better-off. Finally, residuals play different roles across models. While the unexplained sources of inequality tend to favour the worse-off for girls, they seem to favour the better-off for boys.

## Discussion

Our concentration indices show that there is a significant income-related inequality in leisure-time physical inactivity for children, which is higher for girls than for boys. The socioeconomic gradient of physical inactivity in children that is shown in this paper is consistent with previous evidence [11, 12, 14, 15]. Besides, the decomposition analysis also indicates that income and education are the main explanatory factors of inequality. Thus, our results are in line with previous studies that show how family income and parents’ education have a strong influence on the levels of physical activity for children and adolescents [11, 12, 14–16]. Other factors, although less relevant, also show some influence on the propensity to physical inactivity for young people. This is the case of some mental health dimensions and nationality. Previous studies have shown that physical inactivity in adolescents depends positively on depression and negatively on parent support and support from others [20]. Likewise, nationalities other than Spanish reflect a negative association with leisure-time physical activity, particularly for girls. The relationship between physical activity and national origin has been documented before, showing lower activity among foreign-born children, which could be attributed to cultural and normative influences [32] or to neighborhood characteristics [33]. Some evidence also indicates that ethnicity seems to be related to physical activity in adolescents [11]. Even though we only can control for nationality and not for ethnicity, our results would also be pointing out to the influence of cultural issues on the practice of physical exercise. Cultural factors (including beliefs/attitudes about benefits of physical activity), linguistic barriers and several aspects of the neighborhood environment have been proposed as explanatory factors for differences of engagement with physical activity across ethnic-immigrant groups [32]. Although the place of residence shows some influence on the physical exercise developed by Spanish children, we could not check if the specific characteristics of the neighbourhood are associated with children’s behaviour in this respect, as it has been stated elsewhere [13]. Moreover, contrary to what has been suggested by previous studies regarding other health problems of Spanish population, such as adults’ obesity [22], we cannot identify a clear-cut regional pattern referred to the prevalence of leisure-time physical inactivity in children.

This paper has a number of limitations. Firstly, leisure-time behaviour may not adequately represent the global picture of physical inactivity in children. Unfortunately, this is the only information provided by the SNHS, which does not register school activities –not even commuting activities- that may involve the practice of physical activity. Secondly, the paper only analyses ‘extreme’ behaviours, as our dependent variable focus on physical inactivity. However, according to our data, this is not at all an uncommon problem amongst children, as 17 % of the boys and 24 % of the girls do not make any physical exercise in leisure time. Besides, information contained in the SNHS does not allow defining different thresholds of what is considered an adequate level of physical activity according to children’s age, as health authorities recommend [34]. Furthermore, previous evidence has shown that efforts have to be made to promote some physical activity in inactive individuals, as greatest reductions in mortality risk are observed for the lowest activity groups [35]. Thirdly, the choice of the inequality index implies some value judgments. In particular, the use of the Erreygers’ index in this context implies that the most unequal society is defined as a society in which only the richest 50 % of the children is physically active [36]. Nevertheless, after the intense debate about the characteristics and value judgments of the indices’ spectrum [37–40], there is no consensus on the perspective that should be used to evaluate inequalities in health or health-related lifestyles [36]. Fourthly, some relevant determinants of physical inactivity may be excluded from the regressions. For instance, we only include education of the head of the household, because of the impossibility of distinguishing between the educational level of the mother and the father when several adults are present at the household. This fact might explain the magnitude of the residuals in the decomposition analysis. Additionally, there could be some limitations related with the estimation of the household income, although we cannot predict in which direction inequality may be affected by the potential biases. Finally, this is a cross-sectional study, so it is not possible to discuss its findings in terms of causal relationships.

Despite these caveats the results of this paper are important for various reasons. Firstly, we analyse income-related inequalities in physical inactivity for Spanish children. Therefore, we focus on one of the population groups that have been most affected by the economic crisis [41]. We also provide some evidence about the factors that influence children’s behaviour. For the policy maker, understanding the determinants of physical inactivity may help in the design of effective interventions to prevent unhealthy behaviours and, consequently, to prevent health problems. Also, the decomposition approach developed in the paper contributes to better outline interventions aimed at reducing present and future socioeconomic inequalities in health.

## Conclusions

According to our results, there is a significant income-related inequality in leisure-time physical inactivity amongst Spanish children favouring the better-off, particularly for girls. Furthermore, we may conclude that there is an important inequity in the distribution of physical inactivity, since inequalities are mainly explained by social determinants (basically income and education). As physical inactivity in childhood is related to different health problems during adulthood, inequalities here described appear as a plausible cause of health inequalities (and inequities) in adult population. Moreover, the results suggest that public policies aimed at preventing unhealthy habits in childhood and early adolescence, such as physical inactivity, should focus on the most disadvantaged groups in order to reduce the prevalence of those habits and also the socioeconomic gradient in health indicators.

Also, as the influence of many health styles’ determinants significantly differ by gender, this study points out the need of addressing the research on income-related inequalities in health habits from a gender perspective.

## Appendix

**Table 4:** Interval regression estimation for household income

Variables	Coefficient	Robust Std. Error	Z
Constant	242.565	82.572	2.94
Male	226.936	14.045	16.16
Age	39.555	2.444	16.18
Age square	−0.338	0.022	−15.72
Primary education	127.438	16.595	7.68
Secondary education	302.074	23.476	12.87
University education	537.655	27.807	19.34
Employed	477.316	23.389	20.41
Unemployed	−373.483	28.432	−13.14
Social_class_II	−253.399	37.619	−6.74
Social_class_III	−441.163	31.682	−13.92
Social_class_IV	−556.986	33.084	−16.84
Social_class_V	−689.698	31.377	−21.98
Social_class_VI	−789.058	33.356	−23.66
Region_2	325.829	36.075	9.03
Region_3	212.389	35.229	6.03
Region_4	216.793	43.254	5.01
Region_5	−146.155	29.845	−4.90
Region_6	117.912	36.143	3.26
Region_7	−24.572	28.812	−0.85
Region_8	195.251	29.172	6.69
Region_9	236.874	23.729	9.98
Region_10	27.291	22.970	1.19
Region_11	−54.978	29.108	−1.89
Region_12	92.613	32.879	2.82
Region_13	244.037	25.354	9.63
Region_14	134.325	34.378	3.91
Region_15	405.378	39.332	10.31
Region_16	415.354	30.775	13.50
Region_17	161.149	36.176	4.45
Region_18	−125.391	81.089	−1.55
N	15.290		
Sigma (*σ*)	671.655	5.872	
McFadden’s adj. R^2^	0.132		

Independent variables refer to the head of the household. Estimation is performed using sampling weights. Reference categories: female. unschooled or illiterate. inactive. social class I (managers and entrepreneurs with more than 10 workers. and professionals traditionally related to college degrees) and residing in Andalusia
